# Biomimetic materials research, from interest-driven to society-serving: an interview with Shu-Hong Yu

**DOI:** 10.1093/nsr/nwae246

**Published:** 2024-07-17

**Authors:** Weijie Zhao

## Abstract

In August 2016, Shu-Hong Yu's group at the University of Science and Technology of China published an article in *Science*, reporting the first artificial nacre material obtained by imitating the growth process of natural nacre. The artificial nacre has a chemical composition and microstructure highly similar to that of natural nacre, and therefore is both strong and tough. Over the past two decades, Yu's team has been focusing on the field of biomimetic materials. In addition to shells and pearls, their mimetic targets also include wood, animal bone, fish scale, bamboo joint, lotus root silk and others. Their biomimetic materials have excellent mechanical properties and good application prospects in many fields. In this interview with *NSR*, Yu introduced many of his interesting works. His research was initially driven by interest and curiosity, but in recent years, he also hopes to shoulder more social responsibility as a scientist and benefit society with his research outputs.

## Teaser

Over the past two decades, Shu-Hong Yu's team has been focusing on the field of biomimetic materials, with mimetic targets include shells, pearls, wood, bone, fish scale, bamboo joint, lotus root silk and others.

***NSR:*** Can you give us some examples for the application of biomimetic materials?

***Yu:*** Biomimetic material is a basic research discipline with a long history, but is experiencing a new surge of interest and development in recent years. There are many examples of biomimetic applications. Most aircrafts imitate the overall structure of bird wings to enhance balance and reduce airflow resistance; some swimsuits are inspired by shark skin and designed to help the swimmers improve their performance. The all-biomass light-weight high-strength biomimetic materials developed by our group also have good application prospects. They have the potential to replace plastic products in the near future and to accelerate the coming of the post-plastic era.

***NSR:*** What are the all-biomass light-weight high-strength biomimetic materials?

***Yu:*** First of all, the raw materials for the synthesis are all biomass, such as leaves, branches, straws and other waste natural materials. We reduce these biomass materials to basic nanoscale building units, such as cellulose nanofibers, and then assemble them into new composite materials with designed biomimetic structures, in form of films or bulk materials. Such materials have excellent mechanical properties including light-weight, high-strength and high-toughness, and are both degradable and environmentally friendly, thus could potentially substitute plastic in many application scenarios.

## THE MATERIALS FROM YU'S GROUP

***NSR:*** How did you get into the field of biomimetic materials?

***Yu:*** In the early years, I was engaged in the research of liquid phase synthesis of nanomaterials. Then as a Humboldt scholar

**Figure 1.**
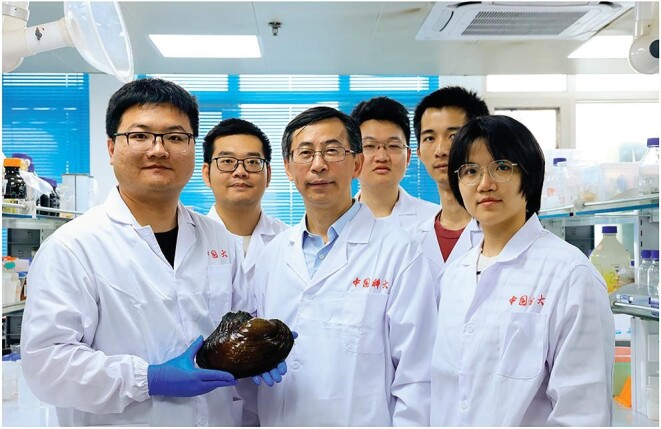
Prof. Shu-Hong Yu (center) and group members (Courtesy of Yu's group).

at the Max Planck Institute for Colloid and Interface, I studied biomineralization. In nature, the formation processes of shells, pearls, as well as bones and teeth of animals, are called biomineralization. These processes often require the participation of biomacromolecules, so that the inorganic components such as calcium carbonate and hydroxyl calcium phosphate can be assembled into ordered microstructures and form biomaterials with excellent macroscopic properties.

After returning to China in 2002, I set up my independent research group and continued with research on the fine regulation of mineral phases and structures. At that time, researchers were focusing on nanoparticles, in most cases, in the form of powders, which could be used as coating materials or additives to improve the performance of materials. However, the production yield of the powders is relatively low, and their delicate structures can only be seen under electron microscopes. So I thought about how we can grow the materials from nanoscale building blocks

**Figure 2.**
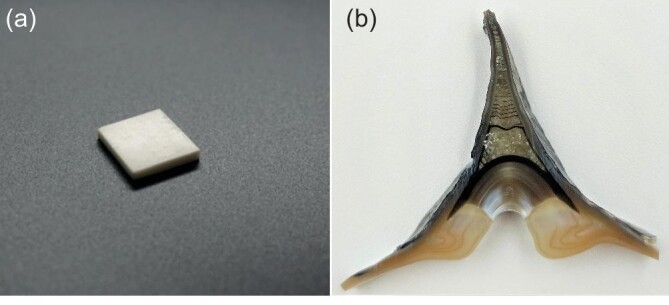
(a) The synthetic nacre fabricated by predesigned matrix-directed mineralization. (b) The hinge cross-section of the freshwater bivalve *Cristaria plicata* (Courtesy of Yu's group).

to macroscopic bulk materials with delicate structures, just like the natural shells, corals and bones.

In 2010, supported by a National Major Scientific Research Program Project on lightweight nanocomposite materials with high strength and toughness, we began to explore how to imitate the structure and growth process of biomaterials, and try to synthesize and grow novel biomimetic materials in a completely artificial environment.

***NSR:*** After the 2016 *Science* article reporting the first artificial nacre material, what progress have you made in the research on sea shells?

***Yu:*** In 2016, my group synthesized artificial nacre in the laboratory for the first time. Natural nacre has a layered bricks-and-mortar structure. Similar to building walls with cement and bricks, the biological macromolecules are secreted to form one layer of ‘cement grids’, in which calcium carbonate grows into ‘bricks’. In this way, the nacre structure is stacked layer by layer. We simulated this process and successfully grew an artificial nacre material with a similar structure.

However, compared with natural nacre, the properties of our synthesized one still lagged behind. My team has been thinking about how to further improve the mechanical properties of the layered biomimetic materials over the years. In 2022, we had new understanding of the toughening mechanism of sea shells, and successfully introduced residual stress into the layered structure of synthesized nacre by adding iron oxide nanoparticles during the mineralization process, which greatly increased the strength and significantly improved the toughness of shell-like ceramics.

In the study of nacre, we also paid attention to the hinge structure connecting the two valves of a shell. Similar to the nacre, the hinge is also composed of calcium carbonate mineral and organic matrix, but the hinge can deform and is resistant to fatigue failure during repeated opening and closing of the shell. I expected the study of the hinge would bring us new inspirations. After more than 10 years’ research, we discovered a deformable bioceramic, and its mechanism of anti-fatigue and deformation based on a preliminary analysis of the microstructure and the motion mechanism of the hinge, which can guide the design and preparation of new anti-fatigue materials in the future. This work was published in *Science* in 2023.

***NSR:*** Besides the shell, what other biological materials have you imitated? How do you choose the imitation targets?

***Yu:*** Our research group has been working on biomimetic materials since 2002. Sea shells have been our long-term research subject. Besides that, since 2017, we begun to analyze and imitate the Bouligand structure that exists widely in nature. The Bouligand structure can be found in the scales of Arapaima, a kind of fish living in the Amazon River basin. This fish skin has a layered structure composed of nanofibers. In each single layer, the fibers are arranged in one direction to form a membrane, and the fibers in adjacent layers are arranged at an angle of 3 to 5 degrees. Thus, the whole structure is spirally stacked layer by layer. Similar structures can also be found in the hammer of a stomatopod, the underbelly of a lobster and other biological materials. We revealed the basic mechanics of the fiber-based Bouligand structure in bamboo joints and other materials, and constructed a series of biomimetic micro-/nano-composites.

Regarding the selection of biomaterials to be imitated, as we mainly focus on the mechanical properties of materials, we usually select biomaterials with superior mechanical properties. We discover and analyze the structure-function relationship of the biomaterials, and then extract the principles that can be applied to the design of biomimetic materials. These novel biomimetic materials exhibit outstanding mechanical properties (strength, toughness, modulus, hardness, fatigue resistance, etc.), and will play key roles in many industrial fields.

***NSR:*** Which of the biomimetic materials synthesized by your group have been or will be used in practice?

***Yu:*** Many materials from our group have great application prospects. Our bone-mimetic material has been applied to repair animal bones and has achieved good results. In 2022, our air purification materials have been used in the production of air purifiers. Our biomimetic poly(lactic acid) coated mica nanosheet has great application potential in food packaging—students of our team won the national gold medal of the ‘Internet+’ innovation and entrepreneurship competition in 2023 by virtue of this research project, which laid a good foundation for the industrialization of this material in the future.

We are actively searching for cooperation with different enterprises and research institutes to explore more application scenarios for our biomimetic materials, including all-biomass fireproof and insulating aerogels, stretchable and non-resilient surgical sutures, biomimetic fireproof panels, biomimetic insulation materials, and others.

The source of innovation lies in the understanding of the peculiar and amazing biomaterials in nature.

—Shu-Hong Yu

## PROSPECT OF BIOMIMETIC MATERIALS

***NSR:*** At present, most biomimetic materials are imitating the structures and mechanical properties of natural materials. Will our imitation expand to more aspects in the future?

***Yu:*** Yes, the imitation of natural materials will certainly expand to more aspects in the future. First of all, we can imitate a wide range of properties, including the chemical, optical and electrical properties of the biological materials. For example, we can design and prepare more sophisticated organic-inorganic hybrid materials by imitating the natural biosynthesis process of inorganic components or small molecular organics.

Secondly, we can also achieve more functions through bio-inspired material design. For example, we can design anti-fouling coatings or drag-reduction materials by imitating the microstructure of shark skin, design materials with special optical effects by imitating the structure of butterfly wings, and design composite materials with unique electrical and thermal properties by imitating the shell structure.

Generally speaking, the imitation of natural materials will be more diversified in the future, and there will emerge many opportunities for innovation, which will provide us with more possibilities to overcome various engineering and scientific challenges.

***NSR:*** What is the most difficult and creative step in biomimetic materials research?

***Yu:*** For biomimetic materials research, the source of innovation lies in the understanding of the peculiar and amazing biomaterials in nature. In my opinion, the most difficult and creative step is to systematically study the structures of biological materials. It needs the most advanced characterization technologies and interdisciplinary methods to accurately extract the design principles of the biological structures, which can then guide the design and synthesis of biomimetic materials.

In addition, interest is the best teacher. It is very important to cultivate students’ curiosity with nature. Scientists should have confidence and judgment to identify their goals. By persistently tackling the key problems, we will eventually accumulate enough experience and achieve success.

***NSR:*** What are the challenges and opportunities in the field of biomimetic materials?

***Yu:*** The challenges mainly come from two aspects. One is the design. Our understanding of natural biomaterials is currently gained through individual case studies, which lacks systematic induction. We have not formed a principle library for biomimetic materials design.

The other one is the scalable preparation. Although many biomimetic materials have been prepared in the laboratory, we still need to solve the problem of scale-up production if we want to really use these materials. There are many problems here, i.e. the performance of the materials may decrease after scale-up, and some materials are not easily produced into certain shapes.

Of course, there are also great opportunities here. In recent years, we have developed more characterization and simulation methods, so that we can systematically study and summarize the ‘structure-component-function’ relationship of biomaterials. For starters, we may find some common rules and characteristics in a class of biomaterial that exists in different organisms.

At the same time, we are also concentrating on the development of large-scale preparation technologies, and cooperating with enterprises to apply our materials in their products.

## PERSONAL OUTLOOK

***NSR:*** In the context of the new era, what changes have you made to your research plan?

***Yu:*** For a long time in the past, my research was mainly driven by interest and curiosity. But in recent years, I am considering more about the social responsibility as a scientist, and often think about what kind of scientific research is most needed by society. We hope to realize the industrialization of our basic research results and truly serve the development of the economy and society, rather than just stopping at the laboratory level. Therefore, in the future, we will set our research directions based upon important practical applications, so that we can create new materials that can really solve practical problems.

***NSR:*** What are your plans and expectations for the next five years?

***Yu:*** In the next five years, under the background of ‘carbon peaking and carbon neutrality’, I will continue to devote myself to the design and development of biomimetic sustainable materials, which will play a very important role in sustainable development. China has abundant biomass raw materials, which are produced by photosynthesis and are inexhaustible green raw materials. Creating new materials based on these renewables is the key way to solve the plastic pollution problem in the post-plastic era. We will continue to work in this direction.

## Notes

Weijie Zhao is an NSR news and science editor based in Beijing.

